# Relationship between housing insecurity, diabetes processes of care, and self-care behaviors

**DOI:** 10.1186/s12913-022-07468-7

**Published:** 2022-01-13

**Authors:** Elise Mosley-Johnson, Rebekah J. Walker, Madhuli Thakkar, Jennifer A. Campbell, Laura Hawks, Sarah Pyzyk, Leonard E. Egede

**Affiliations:** 1 Division of General Internal Medicine, Department of Medicine, Medical College of Wisconsin, 8701 Watertown Plank Rd., WI 53226-3596 Milwaukee, USA; 2 Center for Advancing Population Science, Medical College of Wisconsin, WI Milwaukee, USA

**Keywords:** Housing insecurity, Diabetes, Quality of care, Diabetes processes of care, Diabetes self-care behaviors, Employment status, Disparities

## Abstract

**Background:**

The aim of this analysis was to examine the influence of housing insecurity on diabetes processes of care and self-care behaviors and determine if that relationship varied by employment status or race/ethnicity.

**Methods:**

Using nationally representative data from the Behavioral Risk Factor Surveillance System (2014-2015), 16,091 individuals were analyzed for the cross-sectional study. Housing insecurity was defined as how often respondents reported being worried or stressed about having enough money to pay rent/mortgage. Following unadjusted logistic models testing interactions between housing insecurity and either employment or race/ethnicity on diabetes processes of care and self-care behaviors, stratified models were adjusted for demographics, socioeconomic status, health insurance status, and comorbidity count.

**Results:**

38.1% of adults with diabetes reported housing insecurity. Those reporting housing insecurity who were employed were less likely to have a physicians visit (0.58, 95%CI 0.37,0.92), A1c check (0.45, 95%CI 0.26,0.78), and eye exam (0.61, 95%CI 0.44,0.83), while unemployed individuals were less likely to have a flu vaccine (0.84, 95%CI 0.70,0.99). Housing insecure White adults were less likely to receive an eye exam (0.67, 95%CI 0.54,0.83), flu vaccine (0.84, 95%CI 0.71,0.99) or engage in physical activity (0.82, 95%CI 0.69,0.96), while housing insecure Non-Hispanic Black adults were less likely to have a physicians visit (0.56, 95%CI 0.32,0.99).

**Conclusions:**

Housing insecurity had an influence on diabetes processes of care and self-care behaviors, and this relationship varied by employment status and race/ethnicity. Diabetes interventions should incorporate discussion surrounding housing insecurity and consider differences in the impact by demographic factors on diabetes care.

## Background

Diabetes is the seventh leading cause of death in the U.S. and individuals living with diabetes experience significant economic burden with medical costs being twice as high as those without diabetes [1]. Non-Hispanic Black (NHB) and Hispanic adults are more likely to die from diabetes compared to Non-Hispanic Whites (NHW) and are more likely to develop complications of diabetes and lower limb amputations [2, 3]..

Housing insecurity, defined as having challenges paying rent, doubling up living with friends or relatives, or spending over 50% of household income on housing compounds existing economic burden for individuals with type 2 diabetes mellitus [4–7]. While housing insecurity is correlated to income, a person’s income does not necessarily determine or prevent housing insecurity [5].High housing costs or other expenses in proportion to income, lack of access to affordable housing, number of people supported by income, and substandard/unsafe housing creates conditions for housing insecurity [5]. For individuals with diabetes, housing insecurity interferes with diabetes related expenses and creates a lack of control and consistency necessary to maintain diabetes routines and diets, which are foundational to self-management [8]..

Diabetes processes of care are conducted within the context of the healthcare system and are designed to prevent or delay complications [9]. Diabetes self-care behaviors are often daily self-management routines conducted by individuals with diabetes [1, 9–12]. Both aspects of care are an integral part of comprehensive care for diabetes, and help with preventing diabetes related morbidity and mortality [1, 9–12]. Diabetes process of care and diabetes self-care behaviors are also positively correlated with lower A1C, higher quality of life, and fewer complications [1, 9–12].

Having unmet material needs, including housing insecurity, has been associated with elevated blood glucose levels, increased use of health care resources, and impacts cholesterol, blood pressure, and quality of life in individuals with diabetes [5, 13–18]. Unstable housing in particular has been found to be associated with greater odds of diabetes-related emergency department use, hospitalization, and outpatient visits leading to high health care expenditures [6, 18–21]. Even when environmental and economic challenges are similar, evidence suggests individuals experiencing housing insecurity in public housing have a higher risk of diabetes compared to residents who are stably housed in public housing [22]. Housing insecure individuals need to invest more resources and personal energy into getting around barriers to managing their diabetes, and clinicians find maintaining regular standards of care challenging for housing insecure individuals [23]. However, little is known about the association between housing insecurity and the recommended processes of care and self-care for those with diabetes. In addition, poor diabetes outcomes impact a person’s ability to work and maintain income and savings and wide disparities are noted in both processes of care and self-care behaviors by race/ethnicity. What is not known is if there is a differential relationship by employment or race/ethnicity in the relationship between housing insecurity and diabetes care [24–26]..

This study aimed to address this gap in knowledge by using a nationally representative sample of adults with diabetes to determine if housing insecurity was associated with diabetes self-care behaviors and processes of care, and if the relationship was moderated by employment or race/ethnicity. We hypothesized that housing insecurity is a competing demand and may be associated with a lower likelihood of engaging in diabetes processes of care and self-care behaviors.

## Methods

### Dataset and Sample

The Behavioral Risk Factor Surveillance System (BRFSS) survey is an annual nationwide random-digit-dial telephone survey of the civilian, noninstitutionalized U.S. population 18 years of age or older that collects information on health behaviors, chronic disease and use of preventive services. BRFSS procedures have been reviewed by the Human Research Protection Office of the Centers for Disease Control and Prevention and determined to be exempt research. All methods were carried out in accordance with relevant guidelines and regulations. Analyses are weighted to be representative of this population [27]. States and territories can also elect to administer one of several CDC-approved optional Social Context modules which includes questions on housing insecurity. For the 2014 BRFSS, the Social Context module data was captured from the Midwest (Ohio) and Southern regions (Georgia, Ohio, Tennessee) of the United States. Detailed descriptions of the design and data collection of the BRFSS have been published elsewhere [28]. Diabetes status was determined by response to the question “Have you ever been told by a doctor you have diabetes?”. Those responding “Yes” were considered to be persons living with diabetes and constituted the study sample. We created subsamples for each outcome variable to include participants diagnosed with diabetes who had no missing values on the primary independent variable.

### Outcome Measures

Outcome variables include diabetes processes of care and self-care behavior measures.

Processes of Care:

- *Physician visit.* Having one or more physician visits in the past 12 months was coded dichotomously (yes/no). 7,254 individuals were available for analysis.
- *HbA1c test.* This was assessed by asking “How many times in the past 12 months has a doctor, nurse, or other health professional checked you for ‘A one C’?” The response options were discrete integers. A dichotomous variable categorized as at least one vs. none was computed. 7,071 individuals were available for analysis.
- *Dilated eye exam by provider.* Values were derived from the question “When was the last time you had eye exam in which the pupils were dilated? This would have made you temporarily sensitive to bright light?” Individuals reporting at least one dilated eye exam in the past 12 months were considered to meet quality of care recommendations (3) and were coded as “yes” to create a dichotomous variable. 7,344 individuals were available for analysis.
- *Diabetes Education.* Receipt of education was assessed by asking respondents “Have you ever taken a course or class in how to manage your diabetes yourself?”. A dichotomous variable was created (yes/no). 7,398 individuals were available for analysis.
- *Flu shot.* Receipt of the flu shot was assessed by asking respondents whether they got the flu shot in the past 12 months. A dichotomous variable was created (yes/no). 12,755 individuals were available for analysis.

Self-Care Behaviors.

- *Home blood glucose monitoring.* Respondents were asked “about how often do you check your blood for glucose or sugar? Include times when checked by a family member or friend, but do not include times when checked by a health professional.” A dichotomous variable for frequency of monitoring was created—check sometimes vs. never checks (Yes vs. No). 6,428 individuals were available for analysis.
- *Home foot examination.* Foot care behavior was assessed by asking respondents “about how often do you check your feet for any sores or irritations? Include times when checked by a family member or friend, but do not include times when checked by a health professional.” A dichotomous variable for frequency of foot examination was created—check sometimes vs. never checks (Yes vs. No). 6,280 individuals were available for analysis.
- *Physical activity.* Participants were asked “during the past month, other than your regular job, did you participate in any physical activities or exercises such as running, calisthenics, golf, gardening, or walking for exercise?” An individual was deemed to be physically active if they answered “Yes” to this question. 12,748 individuals were available for analysis.

### Primary independent variable

Housing insecurity served as the primary independent variable and was assessed using the social context module which included the question, “How often in the past 12 months would you say you were worried or stressed about having enough money to pay your rent or mortgage?” Response options were “never,” “rarely,” “sometimes,” “usually,” and “always.” We dichotomized the variable as having housing insecurity “Yes” if they reported that they “always,” or “usually,” or “sometimes” and “No” for “rarely” or “never”.

### Covariates

Inclusion of covariates in the models was based on p<0.25 in bivariate analyses or if considered clinically relevant based on previous literature. Covariates included age (continuous), sex (dichotomized as male or female), race/ethnicity (grouped as Non-Hispanic White, Non-Hispanic Black, or Hispanic/other), education (categorized as less than high school, high school graduate/GED, some college, or college graduate), marital status (dichotomized as married vs. unmarried), household income (categorized as less than $25,000, $25,000-$75,000 or more than $75,000), employment status (dichotomized as employed vs. unemployed) and health insurance status (dichotomized as insured vs. uninsured), and a continuous variable for comorbidity count based on self-report of comorbidities.

### Statistical Analysis

First, sample characteristics were investigated for all individuals in the sample using descriptive statistics to identify the mean and standard deviation for continuous variables and proportions for categorical variables. Second, unadjusted logistic regression models were used to investigate (a) interactions between housing insecurity and employment status, and (b) interactions between housing insecurity and race/ethnicity on each of the diabetes processes of care and self-care behavior outcome measures. Both sets of interaction terms were significant at a *p*<0.05 level for the majority of outcomes so further analyses were stratified first by employment status, and second by race/ethnicity. Therefore, the third step involved running adjusted models for each outcome with housing insecurity as the primary independent variable and adjusting for age, gender, race/ethnicity, education, marital status, household income, employment status, health insurance status and comorbidity count. Five adjusted models (one for each outcome) were run for individuals who were (a) unemployed, (b) employed, (c) Non-Hispanic Whites, (d) Non-Hispanic Blacks, and (e) Hispanic/Other race adults. Adjusted odds ratio estimates for each outcome variable were reported. All analyses were done in Stata/SE 15 [29]. The threshold for identifying statistically significant associations was set at *p* < 0.05. For all analyses, data were weighted to reflect national population estimates and to account for the complex survey design of BRFSS using weights provided in the dataset.

## Results

Table 1 displays the sample demographics for the sample of adults with diabetes who completed the BRFSS survey. 38.1% of adults with diabetes reported housing insecurity. The mean age was 60.5 years and the majority of the respondents were Non-Hispanic White (69.5%). 32.3% graduated from high school or got their GED, 55.4% were married, 82.1% had a household income of $75,000 or less, 67.2% were employed, and 92.6% were insured. The mean comorbidity count was 1.5 (standard deviation of 0.02).

**Table 1:** Sample Demographics for Adults with Diabetes

	Mean (S.E) or %	Weighted sample size
**Housing Insecurity**	38.1% (0.01)	
**Age** (in years)	60.5 (0.19)	
**Gender**		
Male	48.9%	3,543,958
Female	51.1%	3,703,975
**Race**		
Non-Hispanic White	69.5%	4,971,163
Non-Hispanic Black	23.4%	1,674,704
Hispanic/Other	7.1%	506,734
**Education**		
Less than High School	19.7%	1,425,217
High School Graduate/GED	32.3%	2,337,627
Some College	31.4%	2,270,780
College Graduate	16.6%	1,201,093
**Marital Status**		
Married	55.4%	4,005,902
Unmarried	44.6%	3,226,559
**Household total income category**		
Less than $25,000	40.0%	2,484,423
$25,000-$75,000	42.1%	2,617,040
More than $75,000	17.8%	1,107,219
**Employment Status**		
Employed	67.2%	4,860,787
Unemployed	32.8%	2,369,576
**Health Insurance Status**		
Insured	92.6%	6,693,971
Uninsured	7.4%	538,472
**Comorbidity Count**	1.5 (0.02)	

Table 2 displays the unadjusted logistic regression models for the relationship between housing insecurity and diabetes processes of care and self-care behaviors. Individuals who reported housing insecurity had a lower likelihood of having a physician visit in past 12 months 0.67, 95% CI, 0.53, 0.85), an eye exam (0.60, 95% CI 0.52, 0.70), and a flu vaccine (0.70, 95% CI 0.62, 0.78). In addition, individuals reporting housing insecurity were found to have higher odds of daily foot check (1.35, 95% CI 1.13, 1.60) and lower odds of physical activity (1.25, 95% CI 1.05, 1.48).

**Table 2:** Unajusted Relationship between Housing Insecurity and Diabetes Processes of Care and Self-care Behaviors

Outcome Variables	OR (95% Cl)	*p*-value
**Diabetes Processes of Care**		
Doctor Visit	**0.67 (0.53-0.85)**	**0.001**
A1c Check	**0.49 (0.38**-**0.63**)	**0.000**
Eye Exam	**0.60 (0.52-0.70)**	**0.000**
Diabetes Education	**0.83** (**0.72**-**0.95**)	**0.01**
Flu vaccine	**0.70 (0.62-0.78)**	**0.000**
**Diabetes Self-Care Behaviors**		
Blood Sugar Test	**1.25** (**1.05**-**1.48**)	**0.01**
Foot Check	**1.35 (1.13-1.60)**	**0.001**
Physical Activity	**1.25 (1.05-1.48)**	**0.01**

^a^ OR = odds ratio >0.001

^b^Bold text indicates significant a *p*<0.0

Table 3 displays the relationship between housing insecurity and diabetes processes of care and self-care behaviors after being stratified by employment. The relationship between housing insecurity and diabetes processes of care was moderated by employment for seeing a physician in the past 12 months (0.58, 95% CI 0.37, 0.92), having A1c checked in past 12 months (0.45, 95% CI 0.26, 0.78), and having an eye exam (0.61 95% CI 0.44, 0.83) with individuals who were employed less likely to have received those processes of care, but no significant relationship in those who reported they were unemployed. The relationship between housing insecurity and diabetes self-care behaviors was moderated by employment for daily foot check (1.51 95% CI 1.04, 2.19), with individuals who were employed being more significantly likely to check their feet daily, but no significant relationship for those who reported they were unemployed.

**Table 3:** Relationship between housing insecurity and diabetes processes of care and self-care behaviors stratified by employment

	Unemployed		Employed	
	**aOR (95% Cl)**	***p*** **-value**	**aOR (95% Cl)**	***p*** **-value**
**Diabetes Processes of Care**				
Doctor Visit	0.84 (0.58-1.24)	0.391	**0.58 (0.37-0.92)**	**0.010**
A1c Check	1.07 (0.72-1.61)	0.710	**0.45 (0.26-0.78)**	**0.005**
Eye Exam	0.88 (0.69-1.11)	0.308	**0.61 (0.44-0.83)**	**0.002**
Diabetes Education	0.96 (0.77-1.19)	0.707	0.92 (0.67-1.26)	0.588
Flu vaccine	**0.84 (0.70-0.99)**	**0.043**	0.88 (0.68-1.13)	0.326
**Diabetes Self-Care Behaviors**				
Blood Sugar Test	1.04 (0.79-1.36)	0.801	1.03 (0.74-1.44)	0.857
Foot Check	1.14 (0.89-1.46)	0.291	**1.51 (1.04-2.19)**	**0.029**
Physical Activity	0.90 (0.76-1.08)	0.254	0.81 (0.63-1.06)	0.130

^a^Each model is adjusted for demographics, socioeconomic status, health insurance status and comorbidity score

^b^Bold text indicates significant a *p*<0.05 level

Table 4 show the relationship between housing insecurity and diabetes processes of care and self-care behaviors stratified by race/ethnicity. The relationship between housing insecurity and processes of care was moderated by race/ethnicity where Non-Hispanic Whites were less likely to receive an eye exam (0.67, 95% CI 0.54, 0.83) recieve a flu vaccine (0.84, 95% CI 0.71, 0.99), or engage in physical activity (0.82, 95% CI 0.69-0.96), but no significant differences existed for Non-Hispanic Blacks, or Hispanics. Whereas, Non-Hispanic Blacks were less likely to have had a physicians visit in the last year (0.56, 95% CI 0.32, 0.99), with no significant difference for the other racial/ethnic groups.

**Table 4:** Relationship between housing insecurity and diabetes processes of care and self-care behaviors stratified by race/ethnicity

	Non-Hispanic White		Non-Hispanic Black		Hispanic/Other	
	**aOR (95% Cl)**	***p*** **-value**	**aOR (95% Cl)**	***p*** **-value**	**aOR (95% Cl)**	***p*** **-value**
**Diabetes Processes of Care**						
Doctor Visit	0.83 (0.59-1.15)	0.263	**0.56 (0.32-0.99)**	0.049	0.49 (0.16-1.49)	0.212
A1c Check	0.91 (0.62-1.34)	0.626	0.67 (0.37-1.21)	0.184	0.35 (0.09-1.36)	0.130
Eye Exam	**0.67 (0.54-0.83)**	**<0.001**	0.84 (0.57-1.24)	0.384	1.68 (0.88-3.21)	0.113
Diabetes Education	0.90 (0.74-1.11)	0.321	0.94 (0.65-1.34)	0.715	0.95 (0.47-1.89)	0.879
Flu vaccine	**0.84 (0.71-0.99)**	**0.038**	0.84 (0.63-1.12)	0.241	1.25 (0.73-2.16)	0.416
**Diabetes Self-Care Behaviors**						
Blood Sugar Test	0.98 (0.78-1.25)	0.923	1.07 (0.70-1.63)	0.750	1.17 (0.51-2.65)	0.709
Foot Check	1.23 (0.96-1.57)	0.101	1.40 (0.89-2.21)	0.143	1.19 (0.58-2.46)	0.626
Physical Activity	**0.82 (0.69-0.96)**	**0.016**	1.06 (0.76-1.46)	0.741	0.76 (0.45-1.31)	0.323

^a^ Each model is adjusted for demographics, socioeconomic status, health insurance status and comorbidity score

^b^ Bold text indicates significant at the *p*<0.05 level

## Discussion

In a nationally representative sample of adults with diabetes, this study found individuals who reported housing insecurity had a lower likelihood of a number of self-reported diabetes processes of care and self-care behaviors, and this relationship was moderated by both employment and race/ethnciity. The American Diabetes Association (ADA) recommends as part of their standards of care that adults with type 2 diabetes who are meeting glycemic targets visit their physician and receive diabetes education at least annually, receive their annual flu vaccine, and have their A1c checked at least two times per year. The ADA also recommends annual eye exams, however, if there is no evidence of retinopathy for one or more annual eye exams and patient is meeting glycemic targets, then screening every 1–2 years may be considered [9]. Housing insecure individuals with diabetes who reported being employed were less likely to see a physician, have an A1c checked in the past 12 months, or have an eye exam, while unemployed individuals were less likely to receive a flu vaccine. Non-Hispanic White adults with diabetes who reported housing insecurity were less likely to receive an eye exam or flu vaccine or to engage in physical activity, while Non-Hispanic Black adults were less likely to have a physicians visit in the last 12 months.

This paper adds to the current literature by showing how housing insecurity influences processes of care and self-care behaviors and that this relationship differs by employment and race/ethnicity. Findings are in line with previous literature that found unstably housed, low-income families who reported difficulty paying their rent or mortgage were less likely to have a usual source of medical care and more likely to postpone needed treatment than those who have more-affordable housing [5, 23, 30]. Regarding differences by race/ethnicity, Non-Hispanic Blacks and Hispanics have historically been found to be less likely than Non-Hispanic Whites to have a primary care provider for routine preventative care needs and have lower quality of care [2, 30]. Our study found that housing insecure Non-Hispanic White adults were less likely to report receiving an eye exam or flu vaccine when compared to housing secure Non-Hispanic Whites, but that this difference did not exist for Non-Hispanic Black and Hispanic adults, which could indicate a difference in how these processes of care are prioritized based on the level of health care an individual has access to.

Additionally, this study found differences in the relationship between housing insecurity and diabetes processes of care and self-care behaviors by employment status. Specifically, those reporting housing insecurity who were employed were less likely to have a physicians visit, A1c check, and eye exam, while unemployed individuals were less likely to have a flu vaccine. While being employed allows some people to access employer-sponsored health coverage, many low-wage jobs do not come with sufficient sick leave coverage which may create a barrier to seeking health care [31]. It is possible that putting in work hours leaves less time for individuals to make their health a priority. Literature suggests that when people have competing unmet basic needs, they will prioritize needs for food and shelter above health when time and resources are scarce [5]. Interestingly, however, those who were unemployed were less likely to have received a flu shot. Potentially, those who are employed could be more likely to get a flu shot if their employer encourages or requires them to receive one.

One positive finding is that those reporting housing insecurity were more likely to report having received foot care. This could be because foot care is a pillar in clinics that provide care to more vulnerable populations. Foot checks may also be prioritized by those facing housing insecurity because it’s a relatively easy and cost-free self-care activity that can help prevent more serious diabetes complications such as limb amputation and disability. More research into how individuals with diabetes choose to prioritize some self-care activities over others when they are facing challenges meeting basic needs may be warranted.

### Clinical Implications

Socio-economic, social support factors, and clinician promotion of self-care behaviors are considered positive contributors in facilitating self-care activities in patients with diabetes [12]. However, interventions focused only on self-care do not provide clinicians with the tools they need to maintain standard of care in patients who are housing insecure if not also focused on social risks and psychosocial factors [23]. Housing insecurity is known to have a destabilizing effect on all facets of a person’s life impacting their feelings of self-efficacy. As a psychosocial factor, self-efficacy has been found to have a strong association with self-care behaviors and is independently associated with improvements in glycemic status [17]. Self-efficacy interventions for diabetes provide strong evidence for improving health outcomes when deployed by health educators in a clinical setting [32, 33]. Therefore, careful consideration of how to incorporate addressing housing insecurity into interventions aimed to increase positive self-care behaviors and improve self-efficacy are warranted.

The American Diabetes Association recommends that homelessness risk and psychosocial factors (including self-efficacy) are assessed during particular patient encounters and offers tools for these assessments in their standards of care guidelines [9]. Unfortunately, in a nationally represented sample of adults with diabetes who were unstably housed only 2% of participants reported receiving help with housing in a clinical setting. Safety-net clinics that care for the most vulnerable populations often have difficulty putting standard of care guidelines into practice, especially when there is a lack of quality community resources. 6 Housing insecurity could be assessed more systematically via social needs screening tools that incorporate social determinants of health in the Electronic Medical Record (EMR) [34]. Using screening tools in EMRs could be a way to identify when referral to a social worker, patient navigator, intensive case management, wraparound services, and/or clinic-based interventions to address housing. All resources listed have shown improvements in those with chronic disease and could have impact on patients with diabetes facing housing insecurity [9, 35]..

### Limitations

The data is cross-sectional so causation cannot be commented on based on these results. Second, as diabetes was self-reported, those who are living with diabetes but who have not been diagnosed or are unaware of their condition would not be included in the study sample. Third, the analysis was based on data available within BRFSS, so were unable to include details such as how indivduals are receiving diabetes care, duration of time they have been living with diabetes, or regional and local information on urbanization and population density. Future research should explore healthcare datasets that link social context information to healthcare data providing more detailed information on patient level treatment factors. In addition, data that allows for investigation of multiple levels of influence may provide clarity on areas for future intervention. Lastly, the measure of housing insecurity was only assessed using one question and may under-represent the range of experiences surrounding housing insecurity. Unfortunately, housing insecurity has no standard definition and refers to a variety of housing related issues [4, 36]. Without a universal measure and standardized framework for conceptualizing housing insecurity it’s difficult to quantify the scale and severity of the problem. Therefore, estimates may be conservative if housing insecurity was under-represented.

## Conclusions

Self-reported housing insecurity was associated with self-reported diabetes processes of care and self-care behaviors, and this relationship differed by reported employment status and race/ethnicity. To minimize the impact of housing insecurity on diabetes outcomes, housing insecurity questions should be included in standardized assessments used within the clnical setting to further tailor diabetes care interventions to patients and connect those facing housing insecurity to a social service provider with housing resources.

## Data Availability

The datasets analyzed during the current study are available in the Behavioral Risk Factor Surveillance System, BRFSS 2014 survey data and documentation, https://www.cdc.gov/brfss/annual_data/annual_2014.html.

## References

[1] Centers for Disease Control and Prevention. Atlanta, GA: Centers for Disease Control and Prevention, US Department of Health and Human Services. National Diabetes Statistics Report 2020: Estimates of Diabetes and Its Burden in the United States. Available from: https://www.cdc.gov/diabetes/pdfs/data/statistics/national-diabetes-statistics-report.pdf

[2] Canedo JR, Miller ST, Schlundt D, Fadden MK, Sanderson M (2018). Racial/Ethnic Disparities in Diabetes Quality of Care: the Role of Healthcare Access and Socioeconomic Status. J Racial Ethn Health Disparities.

[3] Spanakis EK, Golden SH (2013). Race/ethnic difference in diabetes and diabetic complications. Curr Diab Rep..

[4] Kushel MB, Gupta R, Gee L, Haas JS (2006). Housing instability and food insecurity as barriers to health care among low-income Americans. J Gen Intern Med..

[5] Berkowitz SA, Meigs JB, DeWalt D (2015). Material need insecurities, control of diabetes mellitus, and use of health care resources: results of the Measuring Economic Insecurity in Diabetes study. JAMA Intern Med.

[6] Berkowitz SA, Kalkhoran S, Edwards ST, Essien UR, Baggett TP (2018). Unstable Housing and Diabetes-Related Emergency Department Visits and Hospitalization: A Nationally Representative Study of Safety-Net Clinic Patients. Diabetes Care.

[7] Keene DE, Guo M, Murillo S (2018). "That wasn’t really a place to worry about diabetes”: Housing access and diabetes self-management among low-income adults. Soc Sci Med..

[8] Keene DE, Henry M, Gormley C, Ndumele C (2018). "’Then I found housing and everything changed’: transitions to rent-assisted housing and diabetes self-management. Cityscape.

[9] American Diabetes Association (2021). Standards of Medical Care in Diabetes. Diabetes Care.

[10] Povey RC, Clark-Carter D (2007). Diabetes and healthy eating: a systematic review of the literature. Diabetes Educ.

[11] Odegard PS, Capoccia K (2007). Medication taking and diabetes: a systematic review of the literature. Diabetes Educ.

[12] Shrivastava SR, Shrivastava PS, Ramasamy J. Role of self-care in management of diabetes mellitus. J Diabetes Metab Disord. 2013;12:14. Published 2013 Mar 5. doi:10.1186/2251-6581-12-14 10.1186/2251-6581-12-14 PMC3599009 23497559

[13] World Health Organization. Whiting, D, Unwin, N, Roglic, G. Diabetes: equity and social determinants. Equity, Social Determinants and Public Health Programmes. Published 2010; 77–94.

[14] Agardh E, Allebeck P, Hallqvist J, Moradi T, Sidorchuk A (2011). Type 2 diabetes incidence and socio-economic position: a systematic review and meta-analysis. Int J Epidemiol.

[15] Stahre M, VanEenwyk J, Siegel P, Njai R. Housing Insecurity and the Association With Health Outcomes and Unhealthy Behaviors, Washington State, 2011. Prev Chronic Dis. 2015;12:E109. Published 2015 Jul 9. doi:10.5888/pcd12.140511 10.5888/pcd12.140511 PMC4509099 26160295

[16] Maty SC, James SA, Kaplan GA (2010). Life-course socioeconomic position and incidence of diabetes mellitus among blacks and whites: the Alameda County Study, 1965-1999. Am J Public Health.

[17] Walker RJ, Smalls BL, Campbell JA, Strom Williams JL, Egede LE (2014). Impact of social determinants of health on outcomes for type 2 diabetes: a systematic review. Endocrine.

[18] Walker RJ, Gebregziabher M, Martin-Harris B, Egede LE (2015). Understanding the influence of psychological and socioeconomic factors on diabetes self-care using structured equation modeling. Patient Educ Couns.

[19] Bernstein RS, Meurer LN, Plumb EJ, Jackson JL (2015). Diabetes and hypertension prevalence in homeless adults in the United States: a systematic review and meta-analysis. Am J Public Health.

[20] Axon RN, Gebregziabher M, Dismuke CE (2016). Differential Impact of Homelessness on Glycemic Control in Veterans with Type 2 Diabetes Mellitus. J Gen Intern Med.

[21] Lim S, Miller-Archie SA, Singh TP, Wu WY, Walters SC, Gould LH (2019). Supportive Housing and Its Relationship With Diabetes Diagnosis and Management Among Homeless Persons in New York City. Am J Epidemiol.

[22] Lim S, Liu SY, Jacobson MH, et al. Housing stability and diabetes among people living in New York city public housing, SSM - Population Health. 2020;(11): 2352–8273, 10.1016/j.ssmph.2020.100605. 10.1016/j.ssmph.2020.100605 PMC7287274 32551356

[23] Henry ML, Lichtman JH, Hanlon K, Keene DE (2019). Clinical management of Type II Diabetes among the unstably housed: a qualitative study of primary care physicians. Family Practice..

[24] Breton MC, Guénette L, Amiche MA, Kayibanda JF, Grégoire JP, Moisan J (2013). Burden of diabetes on the ability to work: a systematic review. Diabetes Care.

[25] Mayberry LS, Bergner EM, Chakkalakal RJ, Elasy TA, Osborn CY (2016). Self-care disparities among adults with Type 2 Diabetes in the USA. Curr Diab Rep.

[26] Hill-Briggs F, Adler NE, Berkowitz SA, et al. Social Determinants of Health and Diabetes: A Scientific Review. Diabetes Care. 2020:dci200053. 10.2337/dci20-0053 PMC7783927 33139407

[27] Behavioral Risk Factor Surveillance System. BRFSS 2014 survey data and documentation. Atlanta, GA: CDC. https://www.cdc.gov/brfss/annual_data/annual_2014.html

[28] Behavioral Risk Factor Surveillance System Operational and User’s Guide. Atlanta, GA: U.S. Department of Health and Human Services, Centers for Disease Control and Prevention. Published 2014.

[29] StataCorp (2017). Stata Statistical Software: Release 15. College Station.

[30] Smedley BD, Stith AY, Nelson AR, Institute of Medicine (US) (2003). Committee on Understanding and Eliminating Racial and Ethnic Disparities in Health Care. Unequal Treatment: Confronting Racial and Ethnic Disparities in Health Care.

[31] Antonisse L, Garfield R. The relationship between work and health: findings from a literature review. Kaiser Family Foundation Medicaid Issue Brief. 2018; Retrieved from: https://www.kff.org/medicaid/issue-brief/the-relationshipbetween-work-and-health-findings-from-a-literature-review/

[32] Trinacty CM, LaWall E, Ashton M, Taira D, Seto TB, Sentell T (2019). Adding Social Determinants in the Electronic Health Record in Clinical Care in Hawai’i: Supporting Community-Clinical Linkages in Patient Care. Hawaii J Med Public Health.

[33] Bovell-Ammon A, Mansilla C, Poblacion A, et al. Housing intervention For medically complex families associated with improved family health: pilot randomized trial. Health Aff (Millwood). 2020 Apr;39(4):613-621. doi: 10.1377/hlthaff.2019.01569. PMID: 32250672. 10.1377/hlthaff.2019.01569 32250672

[34] Marks R, Allegrante JP, Lorig K. A review and synthesis of research evidence for self-efficacy enhancing interventions for reducing chronic disability: implications for health education practice (part II). Health Promot Pract. 2005 Apr;6(2):148-56. doi: 10.1177/1524839904266792. PMID: 15855284. 10.1177/1524839904266792 15855284

[35] Fitzpatrick SL, Golden SH, Stewart K (2016). Effect of DECIDE (Decision-making Education for Choices In Diabetes Everyday) program delivery modalities on clinical and behavioral outcomes in urban African Americans with type 2 diabetes: a randomized trial. Diabetes Care.

[36] Cox R, Henwood B, Rodnyansky S, Rice E, Wenzel S (2019). Road Map to a Unified Measure of Housing Insecurity. Cityscape.

